# Miscellanies

**Published:** 1839

**Authors:** 


### MISCELLANIES.

### MR. LISTON ON WET LINT POULTICES.

PERHAPS one of the most striking features in Mr. Liston's "Operative Surgery" is the horror which he entertains of poultices.

We must let Mr. Liston speak for himself. He is talking of wounds.

"In accidental wounds, the part implicated is to be relaxed, and displacement remedied by position; any flap so formed and reverted is retained by the mild and unirritating isinglass-plaster; a stitch may sometimes be put in, or a compress and bandage, applied slackly, may be used for the purpose of keeping the parts somewhat in their relative situation; but any attempt to pull them into close contact, and keep them so, in the hope of union taking place, is futile; in fact, the use of any retentive means ought to form an exception to the general rule which should guide our procedure. The sooner discharge is established the better; lint dipped in cold water may be applied for a few hours till the oozing has ceased, and then with the view of promoting secretion of pus, of thus soothing the painful and uneasy feelings, and of preventing extensive swelling, heat and moisture are substituted. A poultice, always a filthy and uncomfortable application from its weight and stench, may be used; what is much better, equally efficacious, and not liable to objection in any way, is a double piece of lint soaked in hot water, of an agreeable temperature, applied to the part; it is covered by an ample piece of oiled silk, to prevent evaporation, and this dressing, simple enough, but answering every purpose, is removed frequently; the lint may be moistened by merely removing the oiled silk, if the parts are very tender, and if it be not soiled by discharges; all the soothing effect of a poultice is thus produced, without any discomfort. This warm water dressing is light and nice, and it is changed often if there be any unpleasant odour exhaled; it may be medicated with extract of poppies, or salts of opium, or it may be coloured, or have some aromatic added, if the patient does not put faith in simple means. A great deal has been said about water-dressing, and the merit of introducing it; water has been applied to sores from time immemorial. The simple element, water, was supposed to be congenial to wounds and sores; it was used to cool parts. The water-dressing has been used in my hospital and private practice for a long series of years, as a substitute for poultice, as a means of conveying and preserving heat and moisture, on a surface that should secrete pus for its protection, and as an accompaniment of the process of healing by the second intention. A poultice, (the very word is disgustingly associated with putrefaction and nastiness,) has very seldom been employed either in my hospital or private practice, for the last ten or twelve years; in fact, our nurses at the North London have almost forgotten the mode of making the abomination."

### ON THE CAUSES OF SUICIDE. By HENRY WHITFIELD, M.R.C.S. OF ASHFORD.

To ascertain the causes of suicide we must first understand the natural laws of pain and death.

Pain is the sentinel to the body, it warns it of the approach of danger; each organ of the body is guarded by a sensibility peculiar to itself; but feels no more pain than is necessary for its own preservation, and for those organs under its care: thus the skin protects not only itself but the rest of the body; and consequently is endowed with extreme sensibility; it is painfully affected by heat and cold, by cuts and blows, by excess of pressure, and by caustics; the ligaments are insensible to all of these, but give ample warning when over-stretched, as in a sprained ancle; the teeth caution us against acids, although the palate may be agreeably affected; the brain, heart, and stomach have also their respective feelings, and each tells its own tale of distress when abused: much more could be said of the protecting influence of pain, and its nice adaptation to its purpose;* but here it is sufficient to observe that pain is as necessary to protect the body, as food is for its support. Let us now view it in disease; when either the heart, lungs, liver, stomach, or bowels, are attacked with inflammation; it is pain that rouses the sufferer to his danger; and if it gains not his attention, it increases in exact proportion as does the danger, till at last it becomes so intense as to enforce the seeking relief. As the organ recovers, the sufferer is eased of his pangs; but should the art of man be of no avail, is the poor patient to remain in agony? Is he doomed to many years of torment? No—God in his mercy hath ordered it otherwise; death gives its timely aid; the sufferer is at rest. Here again we see there is no unnecessary affliction;† and I wish particularly to draw your attention to the fact; that in all diseases of the body, where death is the natural consequence, the sufferer never attempts self-destruction; it matters not how acute the pain, or of how long duration; or whether he is courageous or cowardly, virtuous or profligate. He may pray for death, but never commits suicide.

Let us now direct our attention to the mind. The mind has its pangs as well as the body, and also has its remedies. When a fellow-creature is deeply distressed from loss of friends, property, or other calamities, as long as he retains his reason, relief is at hand; the word of God teaches him patience under affliction, and points to future bliss, when the present is denied; this, with the sympathy of friends, and other remedies, moral and physical, will restore him once more to cheerfulness: But supposing his calamities to follow so quickly, or from an hereditary, or some physical cause that his reason is overwhelmed, that he is insane, the material organ diseased; where now is the remedy? No religion, no sympathy of friends can enter his mind, he will not so much as listen to his medical advisers; unrestrained by human laws he may be said to be past recovery; here then is no hope; utter ruin. We have seen in diseases of the body that death gives its timely aid; but death comes not here; though now in youth he may live to old age. Why then is the greatest of sufferers denied all consolation, and doomed to many years of torment? It may be said that God afflicts us for our good, to chasten us: every person who is in the habit of visiting the sick has seen the mind improve under the pains of the body; but no man ever witnessed the mind chastened by its own disease; it is impossible.

Why then this exception to the merciful law of death? But is there an exception? Is there ever an exception in the natural laws? Is there not throughout a most perfect consistency? Let us look to facts. Every medical man must have observed that those who are suffering much from a diseased mind have ever a tendency to self-destruction, and this is not confined to those that were daring, and profligate; for the same proneness exists with the mild, timid and religious. She who but a month before would have turned pale at the sight of blood, and have shuddered at the thought of such a deed; will deliberately and firmly raise the deadly weapon against herself when reason has fled, and deep-seated melancholy pervades the soul.

Seeing then that God is ever unwilling that man should be afflicted unneces-

---

* See Sir C. Bell's Bridgewater Treatise.

† See Mr. G. Coombe's "Constitution of Man."

sarily, and that in all diseases where death is the natural consequence, man however daring and wicked never attempts to escape prolonged suffering by suicide: but that in mental disease where death comes not, the sufferer however timid and pious is ever prone to the deed; I am forced to the conclusion that suicide is the natural termination of insanity: and the more I contemplate the subject, the more am I convinced that it is a most wise and benevolent decree of Providence.

One of the advantages arising from this tendency to suicide, is that it has compelled man to legislate for the protection of the invalid, and this protection has proved to be as necessary for the recovery of the mental health, as for the continuance of life.

Before this subject is concluded another question must be discussed; Does man while in possession of his reason commit suicide? This can be best understood by another question. What are the obstacles to the commission of the deed? They are the Christian religion and the instinctive love of life. The Christian religion is of itself ever sufficient with those who during their lives have listened to its ordinances, and have received its support. But can it be expected to be efficacious with those who never read the word of God; whose thoughts are confined to the vain pleasures of the present; the future never entering their minds? No—when therefore one of these worldly men after a life of dissipation and profligacy, has spent his last shilling, and consequently has nothing but extreme poverty with its miseries before him; his reason prompts him to the deed: and will the instinctive love of life be a sufficient barrier to its commission; when we find that on the battle-field, thousands, for glory and promotion, will face the cannon's mouth, or lead a "forlorn hope:" and more especially when we read almost daily of gentlemen standing as a target to be shot at, in preference to giving an apology for a harsh expression uttered during excitement: if rational beings can thus in one act overcome the love of life, and in addition disobey the laws of God, and man, and break through all social, and domestic ties; we may fairly conclude, that the love of life will not prevent the dissipated gamester rushing upon his own sword to rid himself of a world of woes.

But it matters little in a moral view, whether the vicious man has lost his reason at the fatal moment or not; for if my opinion is correct that the Supreme Being in his aversion to unnecessary suffering, gives power to the insane for the escape of many years of useless agony; because they are beyond all consolation and benefit from his revealed word: it is fair to infer that when a man by his intemperance has placed himself in this situation, he has already committed suicide, and each time he deliberately does an act of drunkenness or otherwise inflicts a blow on his brain, he is in some degree guilty of the deed.

In the foregoing observations, I have intentionally avoided entering upon diseases of a mixed character, such as partake both of body and mind, fearing to confuse the subject, as for instance morbid affections of the membranes of the brain are generally disposed to end in death, but from their intimate connexion with the *substance* of the brain, occasionally terminate in suicide.

---

### STUDENTSHIPS IN ANATOMY IN THE ROYAL COLLEGE OF SURGEONS OF LONDON.

The following ordinance can excite but one sentiment, meet but with one judgment—approbation. It is impossible not to perceive that there are men in the present Council of the College who can read the signs of the times, and are inclined to profit by what they read. To those men we appeal with confidence. Let them by a wise profusion, and an economical extravagance, apply some of the revenue of the College to purposes of undoubted professional utility—let them narrow, it may be, their own private income from the College, in order to divert the generous stream into the fair channel of scientific advancement—let them lend their hands to the encouragement of merit in their students and their young members, and further the real interests of all their members—let them shew that they are sensible of the importance of the trust they hold for the surgical body and for surgery—let them do this, (and it should and may be done,) and popularity, not bastard and fleeting and contemptible, but substantial, honourable, glorious popularity, will augment their sphere of usefulness, and swell their measure of dignity.

ORDINANCE.

1. Two studentships in human and comparative anatomy shall be instituted, to be held by each student for the term of three years, at a salary of one hundred pounds per annum.

2. Candidates shall be members of the college, under 26 years of age.

3. The council shall determine, annually, whether one or more of such appointments, shall take place during the current year, and shall notify its resolution by public advertisement.

4. The appointment shall be made in the month of June, or as soon after as possible.

5. The students shall be subject to such duties and restrictions as the council shall from time to time direct; and in case of misconduct shall be liable to dismissal.

REGULATIONS.

1. A report shall be made to the council, in the month of March, of the number of vacancies, or expected vacancies, in these studentships; whereupon the council shall determine whether any, and what number of such vacancies shall be filled up, and shall direct the necessary advertisements.

2. Candidates shall transmit to the secretary, on or before the 1st of May, their applications for the appointment, together with certificates of general good character and of fair acquirements in general learning, signed by two qualified members of the medical profession.

3. A meeting of the museum committee shall be held as soon after the 1st of May as conveniently may be, at which the applications of the persons offering themselves shall be examined, and, if approved, they shall be admitted as candidates.

4. The museum committee shall determine the mode of ascertaining the merits of the several candidates, and shall, after due investigation, report to the council which of the candidates, in their opinion, possesses the highest merit.

5. Students shall attend in the museum daily (Sundays excepted) from ten till four o'clock, and shall be entirely under the direction of the conservators, who shall employ them as they shall see fit; and who have the power of granting leave of absence when they think proper.

6. In case of misconduct or neglect, the students shall be liable to be dismissed at any time by the president and vice-presidents, who are to report such dismissal, with the grounds thereof, to the next meeting of the Council.

May 10th, 1839. EDMUND BELFOUR, Secretary.

*** Upon the present occasion, the applications and certificates must be sent in by the 24th of June next.

---

### New Mode of treating Prolapsus Uteri.

Mr. Benjamin Phillips, as our contemporary* tells us, relates the case of a patient

* Medical Gazette, May 18, 1839.

in the St. Marylebone Infirmary, in whom the infirmity had existed for several years, and who had been unable to bear the irritation occasioned by pessaries of all shapes and of every material employed in supporting the womb. In this case the author succeeded in giving complete relief by destroying a portion of the mucous lining of the vagina by means of strong nitric acid. The contraction which ensued on the separation of the sloughs reduced the vagina to the size of that of a woman who had never borne children.

---

### KLUGE'S TREATMENT OF SYPHILIS.

A correspondent of our contemporary* communicates this for the *benefit* of the English reader.

In every case of primary or of secondary syphilitic affection, Professor Kluge prescribes a quantity of Epsom salts, dissolved in fennel water, sufficient to cause from three to five watery evacuations; and this to be taken every second day during the first week, and every third at a later period. The patient is also usually put on vegetable diet, and kept in a room of moderately warm temperature, and generally confined to bed, especially at first. Local applications are seldom made; and, in gonorrhœa, no local treatment, further than having the parts affected carefully bathed with water, is considered necessary.

In the more obstinate cases of secondary syphilis, instead of salts, the patient takes enough of the decoctum Zittmanni, or of a compound decoction of sarsaparilla (which contains some infusion of senna), to open the bowels two or three times a day. When patients already affected by mercury present themselves, sulphur, or baths of sulphuret of potass, are prescribed as preliminary measures.

With the greatest deference to Dr. Kluge, we should be very sorry to recommend, or imitate his practice. This is the sort of stuff that our young travelling surgeons and physicians fill their heads with. It will never enable them to fill their pockets in this country, we can tell them. The I. M. S. and A. S. S. had far better stop at home, and learn to *cure* their countrymen.

---

### ANTIQUITY OF VACCINATION.

In the Report of the National Vaccine Establishment, it is stated that "the original vaccine lymph, introduced at the Vaccine Establishment by Dr. Jenner, "has passed through nearly a million of subjects *successively*, of whom many thousands have been exposed, with entire impunity, to small-pox in its most malignant form."

On which Mr. Estlin remarks:†—

"Now it is only forty years since the introduction of vaccination, and, however numerous may be the subjects that have been vaccinated at one time from the same individual, the stock of matter at present employed at the National Vaccine Establishment can only have passed through 2080 subjects, even supposing that lymph for subsequent vaccination had been taken every seven days from a fresh subject, without any interruption, from the time when Dr. Jenner first sent it to London. In order that it should pass through a million subjects, the lapse of 19,230 years and 10 months would be required."

As this would carry the origin of vaccination to about thirteen thousand years before the beginning of the world, it is obvious that its antiquity is very considerable. The only wonder is, how the National Vaccine Establishment found it out.‡

---

* Medical Gazette, March 16, 1839. † Ibid. March 9, 1839.

‡ Of course the statement is construed literally; for 500 or 1000 may be, at *the same time*, passing through the vaccine process.

### Medical Valets.

The following advertisement has several times appeared, and many such will no doubt continue to appear in the public journals.

"*To Invalids and Gentlemen.*—Wanted, by a young man, a member of the medical profession, a situation to attend upon or travel with invalids, or gentlemen. Would have no objection to perform the duties of valet. Can give the most satisfactory references as to character, ability, &c.—*Times*, May 27, 1830."

What will the friends of cheap knowledge say to this? They are clamouring against the imposition of tolls on entrance into the profession—they tell us of the hardship, the iniquity, the obstacles they put in the way of poor merit. But high as they may be, cruelly as they may keep out the very deserving *and* the very poor, they are not so enormous, nor yet so cruel, as to bar the ingress of well-informed footmen, and lacqueys of certificated professional acquirements.

Can any one doubt, does any one doubt, that the profession is overstocked? Look down any street, when the gas-lights have superseded the day, and count, if it be possible, the green and red bottles, the blue and bright lamps. Go to any medical advertiser's office—cast the eye on the wrapper of any journal—and the applicants for practice, the hungry expectants of fees, absolutely mob you. How many throbbing heads, how many aching hearts, are engaged in the struggle for bread in the profession—how many repent that their friends were tempted to put them into it—and how many actually engaged in it look with dismay on the annual shoals of young men that enter it. Yet this is the state of things that friends of the profession would perpetuate and extend. What will be the result if the present system is continued? Just such applications as the advertisement we have quoted. Footmen will by-and-bye make it a regular item in their qualifications, that they have been bred as "reg'lar sawbones." We only hope the qualification will be appreciated.

A contemporary asks how it happens that the profession is deemed low in the social scale? The answer is obvious. Admission into it is easy—numbers are consequently admitted—those numbers breed competition—competition knocks down professional remuneration, and gives birth to professional tricks—the arts generated by necessity, the low practices, the shabby appearance bred in the same hotbed of meanness and infirmity, degrade the professors of such a science, and sink it in the world's esteem.

The question is whether the matter shall be made worse, or a vigorous effort shall be attempted for the purpose of making it better. Make it better, say we—make it worse, say the out-and-outers. These Laputan sages care for nothing but their abstract principles. If the profession complain of being pinched and pauperized, "that's low" urge the *feelosofers*. "True *we* like to drive our team—true we have no objection to a good house and a powdered man, (we do not take medical valets on principle)—true we have no objection to get up in the world when we can—but with you it is another matter. You are a liberal profession—you should be above the low desire of ranking with gentlemen, and being thought such—you should feel the " dignity of human nature," and a little reflection might tell you that it is enhanced rather than diminished by a rusty coat, and a scant pair of breeches. Good friends, believe us, you are wrong to reject the great advantages we offer you—the pre-eminent merit of belonging to a profession which looks down upon the vain distinctions of aristocratic pride, and fraternizes with every noble son of a tinker or a tailor, who has scraped up his knowledge by the unassisted force of his own genius."

Medical practitioners of England, see the fate of your sons and your successors. In their hungry contests they may anticipate the fate of the Kilkenny cats, and eat each so effectually as not to leave even a tail behind.

### SWALLOWING PINS AND NEEDLES.

One of the most remarkable cases of this sort ever seen by M. Dupuytren, was "that of a woman, who in consequence of swallowing an incredible number of pins and needles, had become frightfully thin, and was obliged to keep quite still in bed, from the acute pain which was caused on the slightest motion, by the needles and pins, which made their way out from every part of her skin. I opened more than a hundred collections of pus in this woman, at the bottom of which I always found one or two needles or pins. On the surface of this unfortunate person's body there was always fifty or sixty abscesses or tumors caused by the presence of as many of these foreign bodies; which, when added to the number of those which nature was not yet strong enough to drive towards the skin, formed a fearful sum total. It is easy to see that if the presence of a single one of these foreign bodies makes motion difficult and painful, so great a number must bring on a general debility, continued fever, and fatal marasmus; and, in fact, the woman of whom I am speaking died in a hectic state. When her body was opened, several hundred pins and needles were found spread throughout the various organs, the limbs, the cellular tissue, and the muscles; in short, in every part of the body."—*Med. Gaz. Feb.* 23, 1839.

---

### PRACTITIONERS AT HOME AND ABROAD.*

"The Parisian physician may be content with five francs for a service for which the English physician, though perhaps of inferior eminence and talent, would receive his guinea, and all other classes of practitioners may be proportionately remunerated; but the seemingly great disparity is soon compensated by the difference of expense at which the two are obliged to live. If fees are low, so are rents, and taxes, and provisions; and if a man is paid well, he is expected to make a good appearance. By this compensating arrangement, practitioners of both countries probably live up to their income, and occupy about the same general station in society."

We are inclined to doubt this. Many more English physicians and surgeons acquire fortunes than French. We do not mean that the sums saved by the former are only absolutely, but they are also relatively, greater. The guinea fee does more in every way in England than the five franc (a rather high fee) does in France, and every body knows that the French doctor is very much looked down on in society. Too often he lives in a garret, smells of tobacco, and is thought little better than *canaille*.

Some of our contemporaries have talked much of late of the advantage of making British practitioners well acquainted with Continental medicine. What stuff Continental medicine is made of may be judged by the description we shall quote, given us by one who is rather a friend to it. Speaking of the German Journals he says:—

"We see every day, communications to their periodicals of practical medicine, which would scarcely do credit to our nurses: the most crude ideas of the influence of remedies—the exaltation of some of the most inefficient to the rank of the most useful—the praise of many which experience has elsewhere long proved to be inert—and confused and disorderly formulæ—afford a fair idea of what a medical periodical would have been in England two centuries ago. We speak only of the writings of the mass of actual practitioners, more of whom write in Germany than in any other country."—*Ibid.*

And are the French better? It needs no ghost to tell us they are not.

---

* Med. Gaz. June 8, 1839.

